# Complete Genome Sequences of Two Subgenotype 1b Newcastle Disease Viruses Isolated from Sansui Sheldrake Ducks in Guizhou, China

**DOI:** 10.1128/genomeA.01347-16

**Published:** 2016-12-08

**Authors:** Yan Hu, Zhiqiang Duan, Xinqin Ji, Jiafu Zhao, Houqiang Xu, Shunlin Hu, Xiufan Liu

**Affiliations:** a College of Animal Science, Guizhou University, Guiyang, China; b Key Laboratory of Animal Genetics, Breeding and Reproduction in the Plateau Mountainous Region, Ministry of Education, Guizhou University, Guiyang, China; c Key Laboratory of Animal Infectious Diseases, Ministry of Agriculture, Yangzhou University, Yangzhou, China

## Abstract

Here, we report the complete genome sequences of two Newcastle disease viruses, Sheldrake duck/China/Guizhou/01/2016 and Sheldrake duck/China/Guizhou/02/2016, isolated from Sansui Sheldrake ducks in Guizhou Province, China. The genome of the isolates is 15,198 nucleotides in length. Phylogenetic analysis revealed that the isolates are clustered into subgenotype 1b in class I.

## GENOME ANNOUNCEMENT

Newcastle disease virus (NDV), a member of the genus *Avulavirus* within the family *Paramyxoviridae*, has a negative-sense, single-stranded, nonsegmented RNA genome with three sizes (15,186, 15,192, and 15,198 nucleotides [nt]) and encodes at least six viral proteins (1, 2). On the basis of the genomic size and cleavage site motif of the F protein, NDVs can be divided into two distinct classes, I and II. Class I strains have been frequently isolated from wild birds and are low-virulent, while class II strains, including virulent and low-virulent NDVs, have been isolated from wild and domestic birds (3, 4).

Waterfowl are considered to be potential reservoirs of NDV, and both class I and class II NDVs with different genotypes and subgenotypes have been isolated from waterfowl (5–7). Studies have suggested that waterfowl could play an important role in promoting the evolution of NDVs (8, 9). Moreover, some lentogenic strains even have the potential to become virulent through transmission or circulation in poultry populations (10, 11). Therefore, it is necessary to strengthen the surveillance of NDVs isolated from waterfowl to better understand their evolution and genetic characteristics.

Here, we present the genome sequences of two NDV strains, isolated from Sansui Sheldrake duck flocks in Guizhou Province, China, in 2016. The isolates were named Sheldrake duck/China/Guizhou/01/2016 (NDV/GZ01) and Sheldrake duck/China/Guizhou/02/2016 (NDV/GZ02). The complete genome sequences of the isolates were determined by reverse transcription-PCR using 10 pairs of overlapped oligonucleotide primers and direct sequencing. Sequence analysis showed that the full-genome sequences of NDV/GZ01 and NDV/GZ02 are 15,198 nt in length. Compared with the vaccine strain LaSota (GenBank accession no. AF077761), there is a 12-nt (CGGGAAACGGGG) or (CGAGAAACGGGG) insertion in the coding region of the P gene of the isolates, respectively. The nucleotide sequence of the complete genomes and six gene fragments of the isolates all had the highest homology with the sequence of the strain sw/CH/LHLJ/120608 (GenBank accession no. KJ499462). Phylogenetic analysis based on the complete F gene revealed that NDV/GZ01 and NDV/GZ02 were clustered into subgenotype 1b in class I according to the new classification system (12).

Further amino acid analysis of the F and HN proteins showed that the cleavage site of the F protein in NDV/GZ01 and NDV/GZ02 was ^112^ERQERL^117^, which is typical of low-virulence NDV. The HN protein consisted of 616 amino acids (aa), which was the same as sw/CH/LHLJ/120608 but different from the strain duck/Guangxi/1261/2015 (GenBank accession no. KU748779, class I, subgenotype 1c) (585 aa). When compared with consensus amino acid sequences derived from NDV strains of different genotypes and commonly used vaccine strains, the amino acids in the functional domain of the F protein of the isolates showed one mutation (A139S) in the fusion peptide, four mutations (R153K, D170S, T270S, K494R) in the heptad repeat region, and five mutations (V509T, S511A, V513T, F514C, L517V) in the transmembrane domain. In addition, six substitutions (N263R, K333Q, E347D, D349E, I352V, I514V) in the neutralizing epitopes were identified in the HN protein. These results will be helpful for understanding the genetic evolution and molecular characteristics of subgenotype 1b NDVs in ducks.

### Accession number(s).

The complete genome sequences of Sheldrake duck/China/Guizhou/01/2016 and Sheldrake duck/China/Guizhou/02/2016 were deposited in GenBank under the accession numbers KX602322 and KX602323, respectively.
